# Atypical Neuroleptic Malignant Syndrome Without Rigidity During Clozapine Titration: A Diagnostic Challenge

**DOI:** 10.7759/cureus.113175

**Published:** 2026-07-22

**Authors:** Kyaw Thiri Tun, Madhupawani Wijayasuriya Wijayasuriya Arachchige, Shakeel Ahmad

**Affiliations:** 1 Acute Internal Medicine, North West Anglia NHS Foundation Trust, Peterborough, GBR

**Keywords:** atypical neuroleptic malignant syndrome, clozapine-induced nms, clozapine titration, diagnostic challenge in atypical nms, neuroleptic malignant syndrome (nms), rigidity, sepsis mimic

## Abstract

Neuroleptic malignant syndrome (NMS) is a rare but potentially life-threatening complication of antipsychotic therapy, and it may mimic or overlap sepsis, leading to significant diagnostic uncertainty, particularly in the absence of rigidity. Our case was a 64-year-old man with schizoaffective disorder who presented with tachycardia, shortness of breath, and worsening of hand tremors approximately two weeks after the initiation of clozapine. Initial assessment revealed tachycardia and hand tremors with normal muscle tone. Clozapine was withheld due to concern for myocarditis by the psychiatrist. Initial investigations revealed mild elevations of inflammatory markers, and a CT pulmonary angiogram excluded pulmonary embolism.

Within 24 hours, the patient deteriorated, developing high fever, confusion, and hypertension with normal tone and reflexes, prompting sepsis screening, and intravenous empirical antibiotics were commenced. Fever was refractory to antipyretics, and serial blood tests demonstrated a marked increase in creatine kinase (CK) over the subsequent 24-48 hours. Elevated C-reactive protein (CRP) was observed despite persistently normal lactate and negative microbiological cultures including blood, urine, and cerebrospinal fluid. CK elevation was transient, normalizing within five days, while CRP remained elevated for several days. Gradual clinical improvement was noted with supportive treatment.

This case illustrated the rapid clinical deterioration occurring during clozapine titration with concurrent quetiapine therapy, alongside an atypical manifestation of NMS lacking the hallmark rigidity. This atypical presentation posed a diagnostic challenge because the symptoms overlapped with those of sepsis.

## Introduction

Neuroleptic malignant syndrome (NMS) is a rare but life-threatening complication of dopamine antagonists. It is characterized by hyperthermia (>38.0°C {>100.4°F}) on at least two occasions, altered mental status, sympathetic nervous system lability, and hypermetabolism, as well as elevated creatine kinase (CK) [1]. It can, however, present atypically, without some of its classical signs and with consequent diagnostic uncertainty. NMS occurs more commonly in patients taking typical antipsychotics, but it can also occur with atypical antipsychotics. Fever is reported as the most reliable sign, present in 92% of cases [2].

Because of limited evidence, the incidence of NMS is uncertain, but the estimated range is from 0.02% to 3% [1]. The mortality rate is between 8% and 9% according to two large studies. Respiratory difficulties, hyperthermia severity, and older age were associated with increased mortality in those with NMS in one systematic review [1]. However, few significant risk factors for atypical antipsychotic-induced NMS have been identified. The demographic profile of patients who developed NMS with atypical antipsychotics does not seem to differ substantially from that of patients with NMS induced by typical antipsychotics. Male gender, confusion, dehydration, and delirium have been reported as risk factors in one study. The rapid dose escalation of the antipsychotic may also be a risk factor for NMS [3].

NMS is a diagnosis of exclusion, and it is diagnostically challenging as it can mimic sepsis. Acute-phase reactants (APRs) are non-specific inflammatory markers that increase or decrease markedly during inflammation. Changes in acute-phase reactants have been frequently observed in NMS in many studies. There are various mechanisms for the induction of APR response in NMS, such as autoantibody production, virus-drug interaction, heat stress, muscle breakdown, and psychological stress [4]. This case highlights the diagnostic complexity of atypical neuroleptic malignant syndrome during clozapine titration. The temporal dissociation between creatine kinase and C-reactive protein and the presence of possible concurrent infection illustrate the challenges of diagnosing atypical presentations using established diagnostic criteria such as Levenson [5] or DSM-5 [6].

## Case presentation

A 64-year-old man with a background of schizoaffective disorder was referred from a psychiatric facility for the assessment of suspected clozapine-induced myocarditis. On initial presentation, he was alert and orientated. Clinical examination was unremarkable apart from tachycardia (120 beats/minute) and chronic fine tremors affecting both hands. Electrocardiogram showed sinus tachycardia, and initial laboratory investigations revealed mildly elevated inflammatory markers (C-reactive protein {CRP} of 57 mg/L) with a normal white cell count. Troponin was mildly elevated at 22 ng/L, and a repeat measurement was arranged, which was 21 ng/L. Clozapine was commenced two weeks before admission and up-titrated to 350 mg per day (100 mg in the morning and 250 mg in the evening). The scheduled evening dose was withheld by the psychiatric team due to a concern regarding myocarditis and remained suspended following medical review. Other concurrent medications including quetiapine, sodium valproate, and lorazepam were continued.

Within hours of admission, the patient developed acute confusion, pyrexia (39°C), and worsening tachycardia (128 beats/minute). Sepsis screening was done, and broad-spectrum intravenous antibiotics were commenced for probable sepsis. D-dimer was markedly elevated (>3500 ng/mL), prompting a CT pulmonary angiogram, which excluded pulmonary embolism but demonstrated subtle inflammatory changes in the right upper lobe and a reactive right hilar lymph node.

Within the next 24 hours, the patient continued to deteriorate. Reassessment revealed that the patient was confused with persistent fever, high blood pressure, persistent tachycardia, and desaturation to 88% on room air. Respiratory examination was unremarkable, and neurological examination demonstrated fine tremors of both hands, normal muscle tone without rigidity, and normal reflexes. Given the evolving clinical picture, differential diagnosis included sepsis and atypical neuroleptic malignant syndrome (NMS); however, initial normal creatine kinase (CK) and the absence of classical rigidity complicated the diagnosis.

Despite treatment with antibiotics and antipyretics, fever remained refractory. The patient required escalation to the resuscitation area due to ongoing clinical deterioration. Intensive care review suggested that dantrolene should be considered in the event of further deterioration. A single, immediate dose of lorazepam was administered. Supportive management included intravenous fluids, the close monitoring of vital signs, urine output, and renal function, together with empirical intravenous antibiotics while infection remained a differential diagnosis. Clozapine and other antipsychotic medications, including quetiapine and sodium valproate, were held following psychiatric review. The patient maintained a normal urine output and stable renal function. Concurrently, intravenous antibiotics and intravenous fluids were continued. Clozapine was continued to hold, and other antipsychotic medications including quetiapine and sodium valproate were held upon psychiatric review. CK was 268 IU/L, and the vitals became stable, and the patient was later stepped down to the ward.

**Figure 1:**
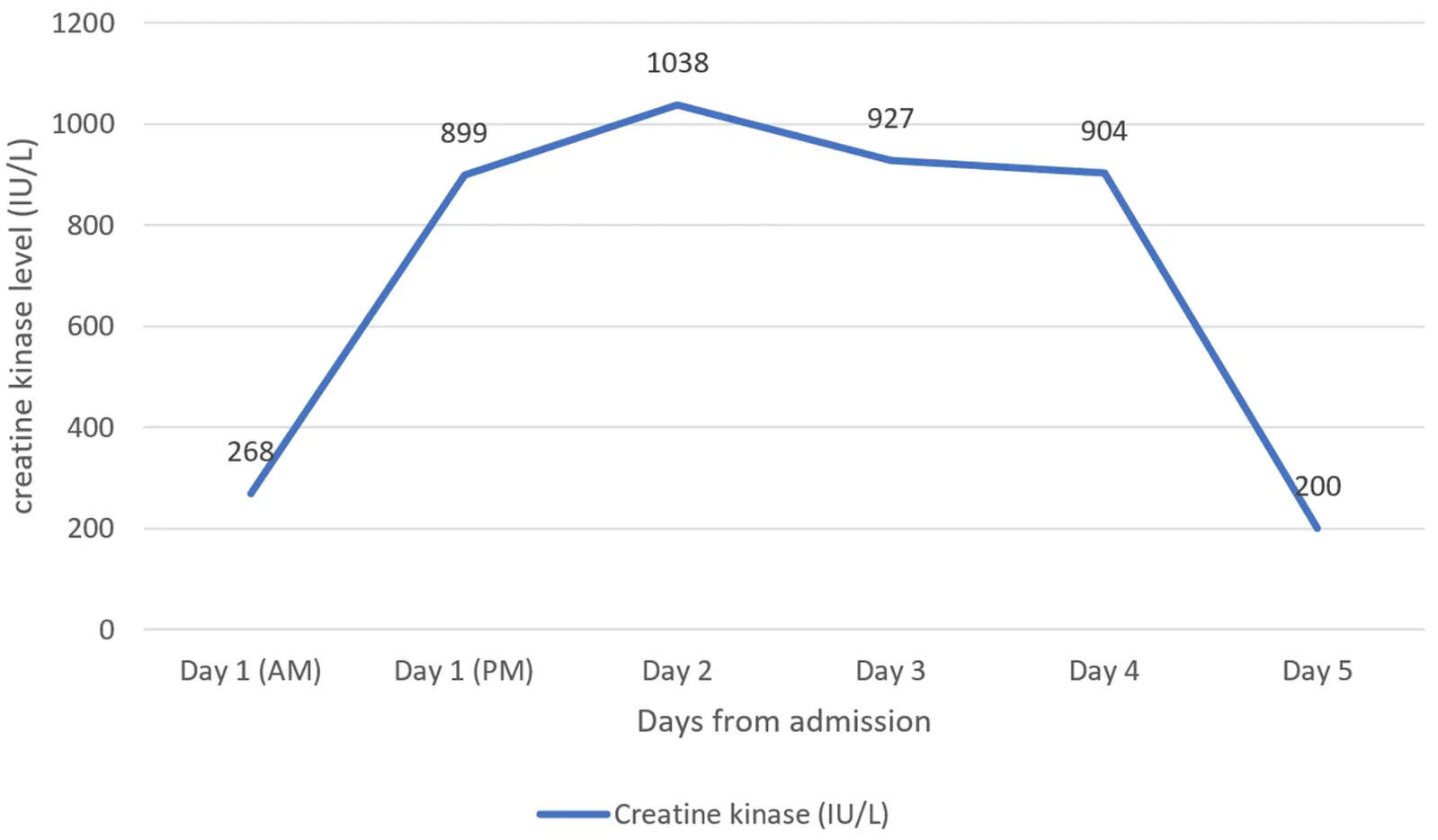
Serial creatine kinase (CK) trend Serial creatine kinase (CK) trend demonstrating an early peak on day 2, followed by rapid normalization by day 5

**Figure 2:**
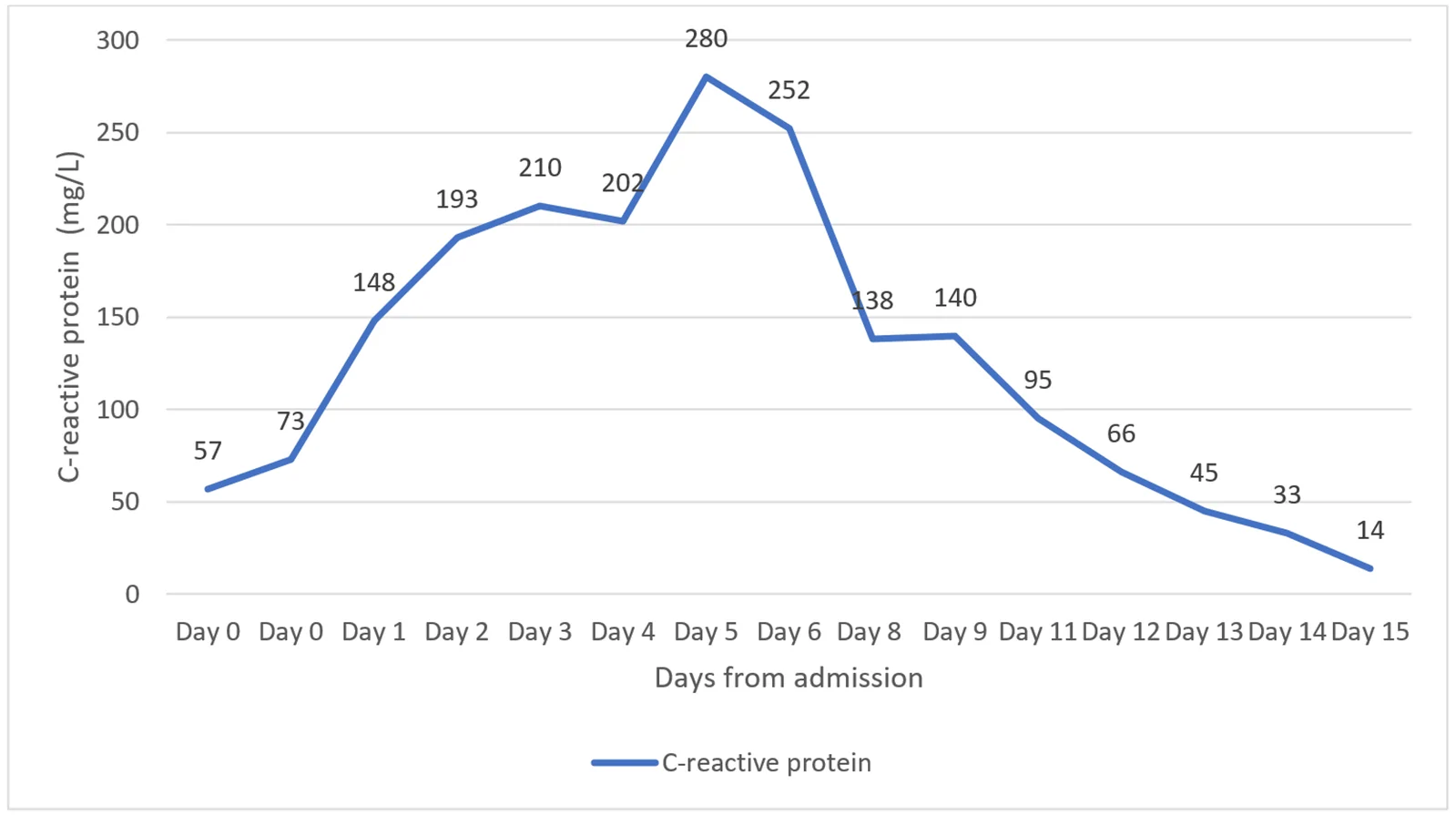
Serial C-reactive protein (CRP) trend Serial C-reactive protein (CRP) trend demonstrating continued elevation despite early CK normalization, with a peak on day 5, followed by gradual decline CK: creatine kinase

On the second day, the patient exhibited further clinical deterioration, characterized by a fever of 39°C, tachycardia of 121 beats/minute, blood pressure of 110/70, and warm peripheries. Patient remained confused and dependent on 2 L/minute of oxygen. Serial CK assessments revealed a rapid elevation from 268 to 1038 IU/L within 24-48 hours after clozapine discontinuation (Figure 1). In contrast, CRP levels demonstrated a gradual upward trend (Figure 2). Further investigations, including a CT scan of the head, revealed no acute findings. Lumbar puncture was performed to evaluate for possible central nervous system infection. The differential diagnoses include atypical neuroleptic malignant syndrome overlapping with sepsis. Despite the presence of fever and elevated inflammatory markers, lactate levels remained normal, and all microbiological tests, including blood, urine, and cerebrospinal fluid cultures, were negative.

CK levels normalized by the fifth day (Figure 1), whereas CRP continued to elevate (Figure 2) despite administration of broad-spectrum antibiotics, with normal lactate and white blood cell counts. Psychiatric consultation suggested a theoretical possibility of cholinergic rebound; however, further investigation into the persistent tachycardia was recommended to exclude causes beyond medication side effects. The patient remained tachycardic, prompting cardiology input. An echocardiogram was performed, which demonstrated a normal ejection fraction with no obvious wall motion abnormalities. Subsequently, the patient developed new-onset atrial fibrillation accompanied by a markedly elevated B-type natriuretic peptide (BNP) level of 7696 pg/mL and was managed with bisoprolol, furosemide, and direct-acting oral anticoagulants. A summary of the clinical timeline is presented in Table 1, while key laboratory findings and investigations are detailed in Table 2 and Table 3, respectively. The patient’s clinical condition gradually improved in parallel with normalization of CRP levels, and he was ultimately transferred back to psychiatric care.

**Table 1:** Clinical timeline CRP, C-reactive protein; CK, creatine kinase; NMS, neuroleptic malignant syndrome

Date/Time Period	Clinical Event
Approximately two weeks before admission	Clozapine initiated and gradually titrated while quetiapine dose was reduced
Hospital day 0 (admission)	Clozapine dose reached 350 mg/day (100 mg, morning; 250 mg, evening). Evening dose was withheld because of concern for clozapine-associated myocarditis following persistent tachycardia. CRP was 57 mg/L, and CK was normal. Serial troponin measurements were unremarkable
Hospital day 1	Developed fever (39°C), confusion, tachycardia, oxygen desaturation, and autonomic instability. Sepsis pathway initiated and broad-spectrum antibiotics commenced. CRP increased to 148 mg/L
Hospital day 2	CK increased from 268 IU/L to a peak of 1038 IU/L. Atypical NMS was considered despite the absence of classical rigidity
Hospital days 2-4	Persistent fever and rising inflammatory markers despite normal lactate and negative microbiological investigations. CRP continued to rise (193 → 210 → 202 mg/L)
Hospital day 5	Peak CRP (280 mg/L) despite the normalization of CK, highlighting diagnostic uncertainty between atypical NMS and concurrent infection
Subsequent course	Echocardiography showed preserved left ventricular function without features suggestive of myocarditis, and CT of the head was unremarkable. CRP gradually normalized with supportive management

**Table 2:** Key laboratory investigations

Investigations	Key Results	Reference Range
Creatine kinase (CK)	Increase from 268 IU/L to peak of 1038 IU/L; normalized by day 5	40-320 IU/L
C-reactive protein (CRP)	Increase from 57 mg/L to peak of 280 mg/L; remained elevated after CK normalization	<5 mg/L
White blood cells	Range from 5.1 to 10.8 × 10⁹/L	4.0-11.0 × 10⁹/L
Lactate	1.2 mmol/L; persistently normal throughout admission	0.6-2.5 mmol/L
Troponin T	22 ng/L with no dynamic rise on repeat testing	<12 ng/L
D-dimer	>3500 ng/mL	0-231 ng/mL
B-type natriuretic peptide (BNP)	7696 pg/mL	<100 pg/mL

**Table 3:** Summary of diagnostic investigations LV: left ventricular

Investigations	Findings
CT pulmonary angiogram	No pulmonary embolism. Subtle inflammatory changes in the right upper lobe and a reactive right hilar lymph node
Echocardiogram	Normal LV systolic function; no regional wall motion abnormalities
CT of the head	No acute intracranial abnormality
Cerebrospinal fluid (CSF) analysis	No evidence of infection
CSF culture	No growth
Blood culture	No growth
Urine culture	No growth

## Discussion

Although NMS is classically characterized by hyperthermia, generalized rigidity, altered mental status, autonomic instability, and elevated creatine kinase (CK), atypical presentations have been increasingly recognized with second-generation antipsychotics, including clozapine [1]. NMS due to atypical antipsychotics such as clozapine can present with atypical features, which can result in diagnostic delay and increase morbidity [7].

Clozapine-associated NMS differs from classical NMS in both clinical presentation and pathophysiology. The rigidity associated with NMS is thought to be due to dopamine D2 receptor blockade in the nigrostriatal pathway. However, the rigidity is often absent with clozapine due to its low affinity for dopamine D2 receptor. Common symptoms include tachycardia and autonomic instability, likely resulting from clozapine acting on adrenergic and muscarinic receptors. CK elevations can be lower and delayed. Atypical features may result from imbalances in neurotransmitters and receptor sensitivity [3,8]. The present case demonstrated several features consistent with previously described atypical clozapine-associated NMS. These atypical features contributed to diagnostic uncertainty during the early phase of illness.

Clozapine-induced NMS is generally less severe than with other antipsychotics and rarely requires ICU admission. The infrequent occurrence of rigidity and extrapyramidal symptoms requires a high degree of suspicion for this diagnosis [3,9]. The mortality rate associated with NMS induced by atypical antipsychotics is reported to be lower than that associated with conventional agents, potentially reflecting increased physician awareness and the prompt initiation of treatment [8]. Clozapine-associated neuroleptic malignant syndrome may occur during the early phase of treatment, with approximately half of reported cases developing within the first two weeks of initiation [8].

In this case, the patient fulfilled the Levenson criteria for neuroleptic malignant syndrome, with two major criteria (fever and elevated creatine kinase) and four minor criteria (tachycardia, blood pressure variability, altered mental status, and diaphoresis) [5,10]. However, the presentation did not fulfil the strict DSM-5 criteria because of the absence of rigidity and leukocytosis [6]. This highlights the diagnostic limitations of existing criteria in atypical clozapine-associated neuroleptic malignant syndrome, where classical features may be incomplete despite a clinically compatible presentation.

In our patient, clozapine was initiated two weeks prior to the presentation and was in the process of dose escalation. Clinical deterioration occurred within 24 hours of admission, with CK levels peaking within the following 24-48 hours. Although concomitant quetiapine therapy may have contributed to overall dopamine receptor blockade, its individual contribution cannot be determined. Recent clozapine initiation and dose escalation were the principal pharmacological changes preceding symptom onset. Although the individual contribution of each antipsychotic cannot be determined, antipsychotic polypharmacy has been recognized as a risk factor for NMS. The clinical features observed in our patient, including the absence of classical rigidity, modest CK elevation, and prominent autonomic features, may overlap with those described in reports of atypical clozapine-associated NMS [11].

Clozapine is the second-generation antipsychotic most consistently associated with withdrawal symptoms. Cholinergic rebound symptoms include nausea, vomiting, diarrhea, headache, agitation, confusion, and diaphoresis. Cholinergic rebound may have contributed to some early clinical features following abrupt clozapine interruption [12]. Some clinical features observed in our patient, including diaphoresis, confusion, autonomic instability, and acute deterioration following clozapine interruption, may overlap with manifestations of cholinergic rebound. However, the subsequent development of persistent hyperthermia, progressive CK elevation, and autonomic dysfunction was more suggestive of NMS than isolated withdrawal phenomena.

NMS is a diagnosis of exclusion, and other causes should be excluded before a diagnosis of NMS is made. Common differential diagnoses are sepsis and drug reactions [1]. The absence of rigidity, initially normal CK, and prominent inflammatory response complicated differentiation from sepsis. Although the peak CK level in our patient was only approximately three times the upper limit of normal, lower and delayed CK elevations have been reported in clozapine-associated NMS compared with classical NMS. Therefore, the relatively modest CK elevation in our patient does not exclude the diagnosis and should be interpreted alongside the overall clinical presentation, serial biochemical trends, and other supportive findings [11]. In our patient, CRP continued to rise despite negative microbiological investigations and persistently normal lactate levels. Furthermore, CK peaked early and subsequently declined, whereas CRP remained elevated for several days. This discordance may contribute to ongoing diagnostic uncertainty and reinforce an initial diagnosis of sepsis [4]. The development of atrial fibrillation and markedly elevated BNP further complicated the diagnostic process and prompted the consideration of alternative cardiac pathology, including myocarditis and acute heart failure. Echocardiography demonstrated preserved systolic function without regional wall motion abnormalities. Whether these findings reflected transient physiological stress secondary to systemic illness or represented an independent process remains uncertain.

Although elevated CRP is frequently interpreted as evidence of infection, published reports suggest that NMS may itself be associated with a systemic inflammatory response characterized by elevated acute-phase reactants, including CRP. Some authors have proposed that acute-phase reactants may be involved in the pathophysiology of NMS, although their precise role remains uncertain [4,13]. In the present case, markedly elevated CRP initially reinforced the suspicion of sepsis and prompted extensive investigation for infection. Although blood, urine, and cerebrospinal fluid cultures remained negative and lactate levels were persistently normal, these findings do not definitively exclude concurrent infection. Therefore, our diagnosis was based on the overall clinical course, serial biochemical trends, the exclusion of alternative causes, and favorable response to supportive management while acknowledging that atypical NMS with concurrent infection remained a diagnostic possibility. Although elevated CRP has been reported in NMS, it is a non-specific acute-phase reactant and should not be interpreted in isolation. In our patient, the persistently elevated CRP together with subtle pulmonary inflammatory changes complicated the diagnostic process because concurrent infection could not be completely excluded. This case highlights that atypical NMS and infection may coexist, and clinicians should avoid diagnostic anchoring in either direction. A notable feature in our patient was the dissociation between CK and CRP. CK peaked within 24-48 hours and normalized rapidly, whereas CRP continued to rise and reached its maximum several days later despite clinical improvement and negative microbiological investigations. The delayed CRP peak despite improving CK further illustrates the difficulty of differentiating atypical NMS from sepsis using inflammatory markers alone.

## Conclusions

This case highlights the importance of considering atypical neuroleptic malignant syndrome in patients receiving clozapine who develop fever, altered mental state, and autonomic instability, even in the absence of classical rigidity. The potential coexistence of concurrent infection and atypical NMS can create significant diagnostic complexity, requiring the careful integration of the overall clinical presentation, serial biochemical trends, and the exclusion of alternative diagnoses. As CRP may be elevated in both atypical NMS and infection, it should not be interpreted in isolation to distinguish between these conditions, and clinicians should consider both sepsis and atypical NMS in the differential diagnosis.

## References

[1] (2025). Neuroleptic malignant syndrome. https://bestpractice.bmj.com/topics/en-gb/990.

[2] Collins A, Davies D, Menon S (2016). Atypical neuroleptic malignant syndrome. BMJ Case Rep.

[3] Sarkar S, Gupta N (2017). Drug information update. Atypical antipsychotics and neuroleptic malignant syndrome: nuances and pragmatics of the association. BJPsych Bull.

[4] Elyasi F, Zarghami M, Fariborzifar A, Cheraghmakani H, Shirzad M, Kazempour F (2023). The diagnostic dilemma in a patient with neuroleptic malignant syndrome during the COVID-19 pandemic: a significant increase in acute phase reactants. Clin Case Rep.

[5] Levenson JL (1985). Neuroleptic malignant syndrome. Am J Psychiatry.

[6] American Psychiatric Association (2022). Diagnostic and Statistical Manual of Mental Disorders, Fifth Edition, Text Revision (DSM-5-TR).

[7] Özdemir İ, Kuru E, Safak Y, Tulacı RG (2018). A neuroleptic malignant syndrome without rigidity. Psychiatry Investig.

[8] Corallo CE, Ernest D (2007). Atypical neuroleptic malignant syndrome with long-term clozapine. Crit Care Resusc.

[9] Trollor JN, Chen X, Sachdev PS (2009). Neuroleptic malignant syndrome associated with atypical antipsychotic drugs. CNS Drugs.

[10] Adityanjee Adityanjee, Mathews T, Aderibigbe YA (1999). Proposed research diagnostic criteria for neuroleptic malignant syndrome. Int J Neuropsychopharmacol.

[11] Belvederi Murri M, Guaglianone A, Bugliani M (2015). Second-generation antipsychotics and neuroleptic malignant syndrome: systematic review and case report analysis. Drugs R D.

[12] de Leon J, Ruan CJ, Schoretsanitis G, De Las Cuevas C (2020). A rational use of clozapine based on adverse drug reactions, pharmacokinetics, and clinical pharmacopsychology. Psychother Psychosom.

[13] Rosebush PI, Anglin RE, Richards C, Mazurek MF (2008). Neuroleptic malignant syndrome and the acute phase response. J Clin Psychopharmacol.

